# Fear of movement and emotional distress as prognostic factors for disability in patients with shoulder pain: a prospective cohort study

**DOI:** 10.1186/s12891-022-05139-6

**Published:** 2022-02-26

**Authors:** Daniel H. Major, Yngve Røe, Milada Cvancarova Småstuen, Danielle van der Windt, Torill Bjugan Sandbakk, Marit Jæger, Margreth Grotle

**Affiliations:** 1 Department of Physiotherapy, Oslo Metropolitan University, Oslo, Norway; 2 Primary Care Centre Versus Arthritis, School of Medicine, Keele University, Keele, Staffordshire UK; 3 Outpatient Clinic for Physical Medicine and Neuropsychology, Helse Møre Og Romsdal HF, Aalesund, Norway; 4 Clinic for Surgery and Neurology, FORMI, Oslo University Hospital, Oslo, Norway

## Abstract

**Background:**

Shoulder pain is a prevalent and often long-lasting musculoskeletal disorder. The overall prognosis of shoulder pain is highly variable with 40–50% of patients reporting persistent pain 6–12 months after consulting a clinician. The evidence for psychological prognostic factors for patients with shoulder pain is inconsistent. Therefore, the objective of this study was to investigate the association between fear of movement and emotional distress at presentation and self-reported disability over one year of follow-up.

**Methods:**

This is a prospective cohort study of consecutive patients referred to secondary outpatient care due to shoulder pain. Consenting patients underwent a physical examination and completed a comprehensive questionnaire at baseline, three months-, and one-year follow-up. Associations between baseline fear of movement (0–10) or emotional distress (1–4), respectively, and patient reported disability measured using Quick Disability of the Arm and Shoulder (QuickDASH, 0–100) over one year were analyzed with linear mixed-effects models (LMM) for repeated measures (baseline, 3 months and 1 year), adjusting for established prognostic factors.

**Results:**

A total of 138 patients were recruited between March 2015 and January 2018, with response rates of 84.7% (*n* = 117) and 79.7% (*n* = 100) at three months and one year, respectively. Adjusted associations revealed that for every point increase in baseline fear of movement, the QuickDASH score increased (worsened) by 1.10 points (95% CI 0.2–2.0) over the follow-up year. For every point increase in baseline emotional distress, the QuickDASH score increased by 19.9 points (95% CI 13.9–25.9) from baseline over the follow-up year.

**Conclusion:**

Higher fear of movement and emotional distress scores at baseline were significantly associated with higher disability over one year in patients with shoulder pain referred to secondary care. Our study indicates that these psychological factors affect prognosis and should be considered by clinicians and researchers working with patients with shoulder pain.

## Background

Shoulder pain is a prevalent and often long-lasting musculoskeletal disorder [1, 2]. The overall prognosis of shoulder pain is highly variable with 40–50% of patients reporting persistent pain 6–12 months after consulting a clinician [3–6]. Shoulder pain disorders often have a multi-dimensional impact on people resulting in pain, activity limitations, social restrictions, sleep disruption, cognitive dysfunction, emotional distress, and other pathophysiological manifestations [7]. The consequences in terms of societal costs are also considerable. A cost-of-illness study from Sweden showed that the mean healthcare cost per shoulder patient was €326 (SD 389) over six months, with physical therapy treatments accounting for 60% of this amount [8]. Conducting prognostic factor research is important to identify and evaluate factors that might be useful as potentially modifiable targets for intervention to improve outcomes, building blocks for prognostic models, or predictors of differential treatment response [9].

Findings from previous prospective cohort studies suggest there is strong evidence that long-term disability in patients with shoulder pain is influenced by duration of symptoms [1, 5, 6, 10–12], baseline pain intensity [4, 5, 10–13], previous shoulder pain episodes [14, 15], baseline disability level [13–17], concomitant neck pain [4, 5, 11] and educational level [14, 17]. Psychological factors are considered by many pain researchers to be the most influential when managing pain [18, 19]. However, the evidence for psychological prognostic factors for patients with shoulder pain is inconsistent. In a systematic review from 2015, the authors found that psychological factors measured at baseline were not associated with pain and disability at follow-up [20]. However, in a more recent systematic review, psychological factors such as high levels of emotional distress, fear of movement and pain, fear-avoidance beliefs, and pain catastrophizing, were reported to significantly influence the perpetuation of shoulder pain intensity and disability [21]. Another recent systematic review of prognostic factors for chronic musculoskeletal pain found supporting reports of an association between a greater degree of fear of movement at baseline and higher levels of pain intensity and disability at follow-up [22]. These findings support the fear-avoidance model, where fear of movement can spark a downward spiral of increased avoidance and emotional distress which in turn leads to increased disability and pain [18, 23, 24].

The fear-avoidance model conveys how a person’s interpretation of their pain may lead to two different pathways [18]. When acute pain is perceived as non-threatening, patients are likely to maintain engagement in daily activities and functional recovery is promoted [18]. In contrast, a vicious circle may be initiated when the pain is catastrophically (mis)interpreted. Pain-related fear, and associated safety seeking behaviors such as avoidance of daily activities and hypervigilance can be adaptive in the acute pain stage, but among patients with persistent pain it may paradoxically worsen the problem [18]. Avoidance can also lead to withdrawal from valued activities and might cause emotional distress (symptoms of anxiety, depression, and somatization), which is known to be associated with decreased pain tolerance [18].

Although there is some support for the suggestion that fear of movement and emotional distress might be barriers to recovery, the authors of the above-mentioned systematic reviews concluded that more prospective cohort studies investigating fear of movement and emotional distress as prognostic factors are needed. The overall quality of the evidence was very low [21, 22] and none of the studies included in the systematic review investigating fear of movement as a prognostic factor included only patients with shoulder pain [22]. Therefore, much uncertainty exists regarding the prognostic value of fear of movement and emotional distress for patients with shoulder pain. The objective of this prospective cohort study was to investigate the association of two psychological factors—fear of movement and emotional distress—at baseline with self-reported disability over a follow-up period of one year among patients referred to an outpatient hospital clinic due to persistent shoulder pain. We hypothesized that a higher fear of movement score and a higher emotional distress score at baseline would be associated with a higher disability score over the year of follow-up.

## Methods

### Study design and setting

This study is a prospective cohort study of consecutive patients referred to a secondary outpatient clinic at Aalesund Hospital (secondary care) in Norway due to shoulder pain, between March 2015 to January 2018. This study was reported according to the Strobe Statement for cohort studies [25] and the REMARK checklist [26], and is based on recommendations from the PROGRESS framework for prognostic factor research [9, 27]. The study was classified as a quality assessment study by the Norwegian Regional Committee for Medical Research Ethics (reference no. 2014/1634/REK vest) and was approved by the local hospital ethical committee (“Personvernombudet”) which is organized under the Norwegian Social Science Data Service (NSD) (reference no. 2017/1166.). In line with the Helsinki declaration, all patients signed a written, informed consent form. The included patients completed comprehensive questionnaires at the outpatient clinic at baseline, 3-months, and 1-year follow-up. The funders did not play any role in the designing, conducting, or reporting of this study.

### Participants

Patients were eligible for inclusion if they presented with a painful shoulder condition, were age 18 years or older, and had a sufficient command of the Norwegian language. Patients were excluded if they had shoulder pain due to other disease (such as systematic disease or cardiac disease), generalized pain, symptoms of cervical spine disease, serious psychiatric disorder, or had had surgery on the affected shoulder within the last 6 months.

### Clinical procedures

#### Diagnostic assessment

The diagnostic assessment was conducted by a specialist in physical medicine and a physical therapist. The assessment was based on a standardized questionnaire, case history, a clinical examination, and sometimes supplementary examinations as well (e.g. magnetic resonance imaging, X-ray). The patient case was discussed in an interdisciplinary team consisting of the physical medicine specialist, physical therapist, and an occupational consultant, and, if necessary, the patient was referred to an orthopedic or other specialist.

### Management

The included patients underwent individualized management which reflected usual practice at the outpatient clinic at Aalesund Hospital. After the assessment, patients were given advice from the interdisciplinary team, based on a biopsychosocial approach. Some patients were offered individual and/or group treatment provided by the team at the outpatient clinic, and some were referred back to primary care clinicians. The main content of the hospital treatment was a cognitive-oriented physical therapy consisting of patient education and supervised shoulder strengthening and mobility exercises, and, in some cases, consultation with the occupational consultant. All patients were invited to a 3-month re-assessment at the hospital.

### Outcome

The primary outcome of the study was the Quick Disability of the Arm, Shoulder and Hand Questionnaire (QuickDASH) [28]. The QuickDASH is a region-specific patient rated questionnaire. Eleven items, covering six domains (daily activities, symptoms, social function, work function, sleep, and confidence) are scored on a numerical rating scale between one (no difficulty) and five (unable). The summed score is expressed as a score where zero represents no disability and 100 represents maximum disability. Previous research has demonstrated good responsiveness [28–31] and good validity, reliability, and precision of the QuickDASH [32]. The minimal important change (MIC) for the QuickDASH has been estimated as an improvement of ≥ 10.8 points when assessed using this specific sample [31].

### Potential prognostic factor measurements

Fear of movement was assessed using one question at baseline [33]: “How much ‘fear’ do you have that these complaints would be increased by physical activity? (scores range from 0 = no fear, to 10 = very much fear). Fear of movement (kinesiophobia) is defined as an excessive, irrational, and debilitating fear of carrying out a physical movement due to a feeling of vulnerability to a painful injury or reinjury [34].

The Hopkins Symptom Checklist-25 (HSCL-25) [35] was used as a measure of emotional distress. The questionnaire aims to assess symptoms of anxiety, depression, and somatization. HSCL-25 is a shorter version of the Symptom Checklist 90 (SCL-90) and consists of 25 items that are rated from 1 (not at all) to 4 (very much). The score was obtained by averaging the scores, and ranges from 1 and 4. A maximum of five missing items was accepted. A higher average score indicates a higher level of emotional distress. The Norwegian version of the HSCL-25 has been used in several studies of musculoskeletal pain [14, 36–38].

### Confounders

As recommended in the PROGRESS framework [9, 27], we adjusted for prognostic factors that have consistently been shown to be associated with the outcome (disability) among patients with shoulder pain and standard demographic factors: age, sex, body mass index (BMI), and educational level [14, 17], duration of symptoms [1, 5, 6, 10–12], and baseline disability score [13–17]. We did not adjust for baseline pain intensity because the domain is covered in the baseline disability score (QuickDASH).

### Statistical analysis

All participants providing baseline data on potential prognostic factors and the outcome (QuickDASH) at baseline and at 3-months or 1-year follow-up were included in the analysis.

Descriptive data were presented as counts and percentages for categorical data, and means and standard deviations or medians and interquartile ranges for continuous normally distributed data and data with skewed distribution, respectively (Table 1).

**Table 1:** Baseline characteristics of the study sample

Characteristics	Completed all follow-ups (*n* = 101)	Did not complete 3-months follow-up (*n* = 15)	Did not complete 1-year follow-up (*n* = 22)
Age *(yr), median (IQR)*	48.5 (16)	42 (14)	42 (19)
Sex, women *n *(%)	75 (74.3)	11 (73.3)	13 (59.1)
BMI, *median (IQR)*	22.2 (5.1)	21.3 (3.0)	22.0 (5.4)
Shoulder diagnosis *n *(%)			
Subacromial pain	67 (66.3)	10 (66.7)	13 (59.1)
Adhesive capsulitis	12 (11.9)	1 (6.7)	4 (18.2)
Myalgia	3 (3)	2 (13.3)	1 (4.5)
Other specific shoulder conditions	12 (12.9)	1 (6.7)	3 (13.6)
Other unspecified shoulder conditions	6 (6)	1 (6.7)	1 (4.5)
Duration of symptoms, *n *(%)			
< 1 month	-	-	-
1–3 months	-	-	-
4–12 months	30 (30)	3 (20)	6 (30)
< 12 months	70 (70)	12 (80)	14 (70)
Educational level, *n (%)*			
Elementary school	14 (14.1)	-	2 (9.5)
High school	40 (40.4)	7 (46.7)	9 (42.9)
Higher education < 4 years	30 (30.3)	4 (26.7)	4 (19)
Higher education ≥ 4 year	15 (15.2)	4 (26.7)	6 (28.6)
Pain intensity, *median (IQR)* (0–10)	5 (3)	6 (3)	5.5 (2.3)
Emotional distress^a^, *median (IQR)* (1–4)	1.4 (0.6)	1.3 (0.6)	1.4 (0.9)
Fear of movement^b^, *median (IQR)* (0–10)	5 (6)	3 (5)	3.5 (5.5)
Disability^c^, *median (IQR)* (0–100)	38.6 (25)	34.1 (17.7)	35.2 (23.9)

^a^Measured by Hopkins Symptom Checklist-25: range 1 (not at all) to 4 (very much)

^b^Measured using one question: “How much ‘fear’ do you have that these complaints would be increased by physical activity?” (scores range from 0 = no fear, to 10 = very much fear)

^c^Measured using QuickDASH: range 0 (no disability) to 100 (maximum disability)

Missing data: No missing data on sex, diagnosis, emotional distress, and disability. Missing data was limited to 1.5% for age, 1% for duration of symptoms, 4% for pain intensity and fear of movement

To estimate the strength of associations between the two potential prognostic factors (fear of movement and emotional distress) and the outcome (QuickDASH score at baseline, 3-months and 1-year follow-up), linear mixed-effects models (LMM) for repeated measures were fitted using an unstructured covariance matrix. The models for the outcome consisted of 3 fixed effects: measurement occasion (time), fear of movement or emotional distress, and the interaction term of time and fear of movement or distress. All measured time points of the outcome variables were considered and the LMM approach therefore adjusts for baseline differences. To test whether potential confounders influenced the results, LMM were adjusted for age, sex, BMI, educational level, duration of symptoms, and disability. Effect sizes of the adjusted associations were also calculated using the formula: d = mean change per point/SD. We also conducted a sensitivity analysis including only the patients with subacromial pain (*n* = 89). The normality assumptions for residuals were assessed by visual inspection (histogram and QQ plots). We used scatter plots to investigate whether the associations between potential prognostic factors and the outcome were linear.

Statistical significance was inferred when the *p* value was < 0.05. SPSS (version 26, IBM, Armonk, NY, USA) and STATA (Version 16.0 StataCorp LP, College Station, TX, USA) was used to perform all analyses.

## Results

**Fig. 1:**
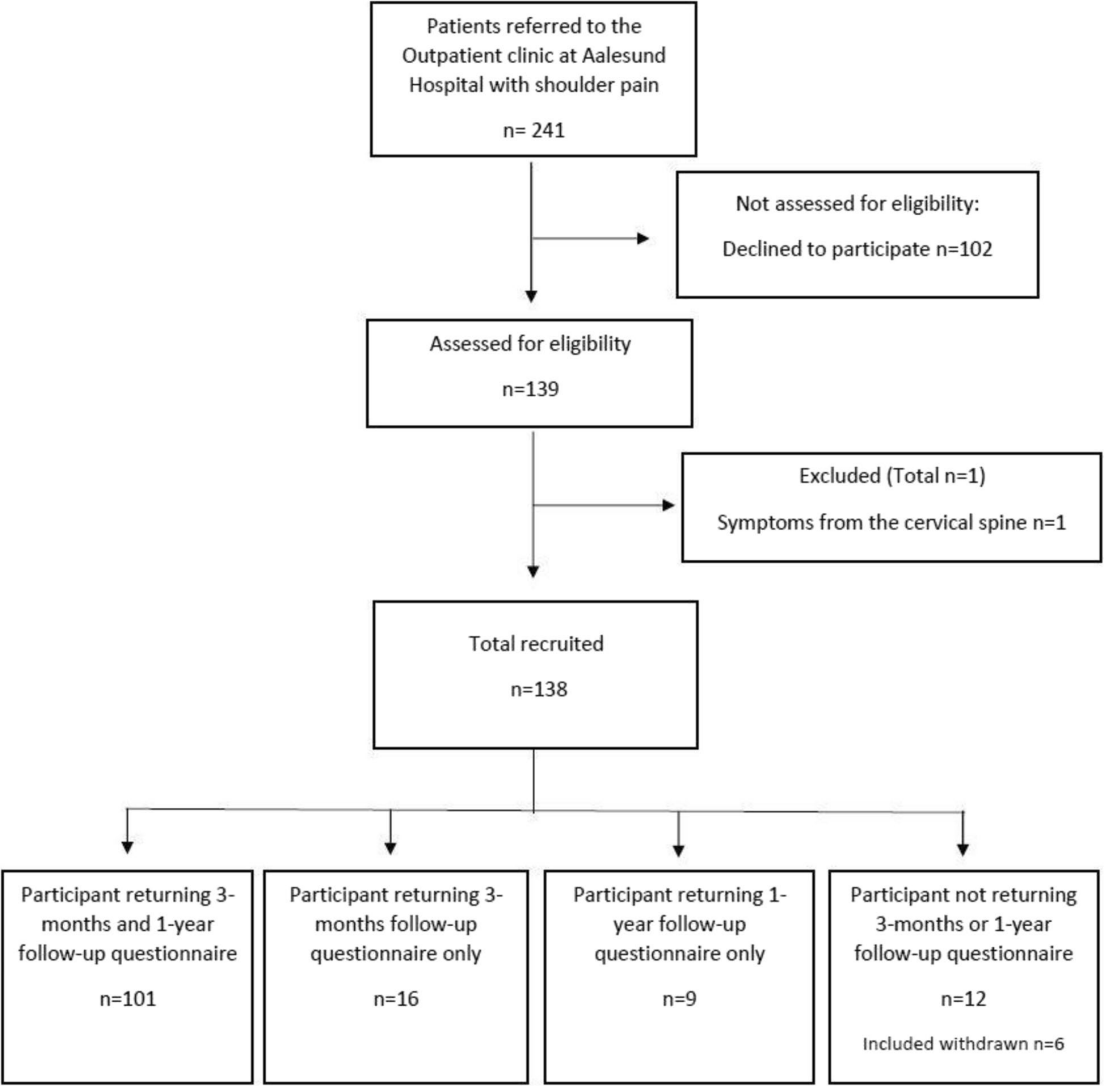
Flow diagram of participants

A total of 138 patients referred to secondary care at the outpatient clinic between March 2015 and January 2018 consented to participate in this study. The flow of participants is depicted in Fig. 1. Six patients withdrew their consent. The response rate at 3 months and 12 months was 85% (*n* = 117) and 80% (*n* = 110), respectively. The baseline characteristics of the included participants are provided in Table 1. Patients who completed all follow-up questionnaires and patients who did not respond to 3-months and 1-year follow-up were similar on most baseline characteristics, but the patients who did not respond to either 3-months follow-up or 1-year follow-up were slightly younger, had lower disability and fear of movement scores, and had slightly higher education.

The included patients underwent a median of 8 (range 1–36) treatment sessions with a physical therapist during the 1-year follow-up period. The patients reported that the main components of the physical therapy management were supervised exercise (74.2%), information/advice (68.2%), group-based exercise (12.1%), stretching (14.4%), and massage/manual therapy (6.1%). Eleven patients (9.1%) reported that they received analgesic medicines during the 1-year follow-up. The mean change from baseline to 3-month follow-up (*n* = 117) was 10.2 QuickDASH points (95% CI 7.9–12.6), and at 1-year follow-up (*n* = 110) it was 13.3 QuickDASH points (95% CI 10.3–16.4). The proportion of participants reporting an improvement corresponding to a minimal important change of ≥ 10.8 QuickDASH points at 3-month and 1-year follow-up was 43.2% and 57%, respectively.

As displayed in Table 2, the adjusted associations from the linear mixed-effects models revealed that for every point increase in fear of movement at baseline, the QuickDASH score increased (worsened) by 1.10 points (95% CI 0.2–2.0) over the year of follow-up. There was no statistically significant interaction between time and fear of movement (*p* = 0.059). The calculated effect size for the adjusted association between fear of movement and QuickDASH score over the follow-up year was 0.24. Adjusted associations from the linear mixed effects models revealed that for every point increase in emotional distress at baseline the QuickDASH score increased (worsened) by 19.9 points (95% CI 13.9–25.9) over the 1-year follow-up. The interaction between time and emotional distress was not statistically significant (*p* = 0.601). The calculated effect size for the adjusted association between emotional distress and QuickDASH score over the follow-up year was 0.65.

**Table 2:** Estimates from linear mixed effects models for the association between fear of movement and emotional distress, measured at baseline, and disability score over the follow-up year

	QuickDASH score (0–100)			
	Unadjusted mean change (95% CI)	*p*-value	Adjusted mean change (95% CI)^a^	*p*-value
Fear of movement (0–10), per point^b^	1.14 (0.29–1.98)	0.01	1.10 (0.20–2.00)	0.02
Emotional distress (1–4), per point^c^	16.93 (11.31–22.55)	< 0.001	19.89 (13.86–25.92)	< 0.001

^a^adjusted for age, sex, BMI, educational level, baseline disability, and duration of symptoms

^b^Measured using one question: “How much ‘fear’ do you have that these complaints would be increased by physical activity?” (scores range from 0 = no fear, to 10 = very much fear)

^c^Measured using the Hopkins Symptom Checklist-25 (scores range from 1–4, 4 = maximum)

In a sensitivity analysis including the patients with subacromial pain only (*n* = 89), results were similar to those in the main analyses: the adjusted associations from the linear mixed-effects models revealed that for every point increase in fear of movement, the QuickDASH score increased (worsened) by 0.64 points (95% CI -0.77 to 2.05) over the year of follow-up. Adjusted associations from the linear mixed effects models revealed that for every point increase in emotional distress at baseline the QuickDASH score increased (worsened) by 22.5 points (95% CI 18.3–26.7) over the 1-year follow-up among the patients with subacromial pain. The interaction between time and the prognostic factors under investigation was not statistically significant for any of the sensitivity analyses.

## Discussion

In this study we found that there was a significant positive association between fear of movement score at baseline and disability score over a 1-year follow-up, which remained after adjustment for age, sex, educational level, BMI, duration of symptoms, and time. A higher emotional distress score at baseline was associated with a higher disability score over a 1-year follow-up, including after adjustment for the above-mentioned covariates.

Emotional distress is increasingly recognized as an important prognostic factor among patients with shoulder pain [21]. Our study adds to the growing body of literature and the results from our study are consistent with those of prior systematic reviews showing that higher emotional distress at baseline is associated with worse self-reported disability and higher pain intensity at follow-up among patients with shoulder pain [21] and musculoskeletal pain [39]. Furthermore, the effect size of 0.65 reported in our study indicates that emotional distress had a moderate effect on disability over the follow-up year. However, it is important to consider that it is unknown whether emotional distress is a causal factor or simply a consequence of shoulder pain. More research is needed to suggest that management should target emotional distress. Nevertheless, the findings from a systematic review and meta-analysis on neck and back pain showed that emotional distress mediates the relationship between pain and disability and therefore concluded that emotional distress might be an important target for treatment [40]. A potential explanation for the association of emotional distress with disability over the 1-year follow-up is that emotional distress is known to be associated with decreased pain tolerance [18]. Emotional distress can also affect perceived self-efficacy and reduce a patient’s ability to self-manage their condition [19, 41], which is important because exercise programs are predominantly performed at home. A post hoc analysis, requested by a reviewer, revealed a statistically significant (*p* =  < 0.001), moderate positive correlation (Spearman’s *r* = 0.42) between baseline disability and baseline emotional distress.

Although the QuickDASH does include domains such as sleep and confidence in being able to perform activities, these items only overlap to a small degree with those included in the HCSL-25. The QuickDASH focuses specifically on the impact of shoulder-upper limb problems on activities and pain, whereas the HCSL-25 measures generic symptoms of anxiety, depression, and somatization. The QuickDASH has been validated against SPADI, showing a strong correlation (*r* = 0.81) [42]. The cross-sectional associations with emotional distress are weaker, confirming that they measure different constructs. Because we adjusted for baseline levels of disability in the LMM, the association between baseline levels of emotional distress with shoulder disability over the one-year follow-up is adjusted for the influence of baseline disability scores.

Similarly, the hypothesis that there was a significant positive association between fear of movement and disability was confirmed. This finding is in line with two other studies suggesting that fear-avoidance beliefs are associated with poor shoulder function in patients with subacromial pain [43, 44]. A systematic review also found that a greater degree of fear of movement at baseline is a significant prognostic factor for the progression of disability over time among patients with chronic musculoskeletal pain [22]. As theorized by the fear-avoidance model, fear of movement can lead to a downward spiral of increased avoidance, disability, and pain [18, 23, 24]. One potential explanation for the present finding is that fear of movement might impose a barrier to performing strengthening exercises. Exercise was a central component of the physical therapy management for these patients and this might have resulted in reduced adherence to treatment and the perseverance of a negative experience with pain [18]. However, the effect size of 0.24 reported in our study reflects that fear of movement at baseline only had a small effect on disability over the follow-up year.

### Implications for practice and research

Key psychosocial obstacles to recovery are becoming clearer, which puts us in a better position to advocate which factors should be the focus for assessment and treatment [45]. Because both fear of movement and emotional distress were significantly associated with the QuickDASH score over the follow-up year, we suggest that patients with high scores at baseline might benefit from psychologically informed physiotherapy to address these psychological obstacles to recovery. Foster and Delitto suggested that identifying and addressing fear of movement might be relatively easy to incorporate into standard physiotherapy practice, while eliciting and addressing emotional distress might require additional training for physical therapists [45]. Management by a multidisciplinary team including health care practitioners with psychological expertise should also be considered as this might confer better outcomes for patients with a high emotional distress score at baseline [46]. Our study indicates that fear of movement and emotional distress affect prognosis in patients with shoulder pain and should be considered by the clinicians and researchers who work with them.

### Strengths and limitations

A strength of this prospective cohort study was that we conducted a sound statistical analysis based on recommendations from the PROGRESS framework [9, 27] and used a validated shoulder-specific outcome measure (QuickDASH). This study also has some limitations. First, the limited sample size and the heterogenous sample including different subgroups of patients with shoulder pain (subacromial pain, adhesive capsulitis, myalgia, and other specified and unspecified conditions) resulted in wide confidence intervals for our estimates, reflecting limited precision [25]. The estimates and their confidence intervals would be more precise if more patients were included. Second, fear of movement was measured using only one question. This question has a similar predictive ability to the Tampa Scale for Kinesiophobia among people with sciatica, but has not been validated for patients with shoulder pain [33]. Third, the patients were recruited from a secondary care outpatient clinic at the hospital in Aalesund (Norway) which might limit applicability of the results to other clinical settings, including primary care. The patients who dropped out from our study were slightly younger, had lower disability and lower fear of movement scores, and had slightly higher education than the patients that were successfully followed-up. Therefore the results might not be generalizable to those patients who left the study, who are also likely to have a better prognosis. Finally, because treatment was not described in detail, we were not able to adjust for the treatment the patients were given in the linear mixed-effects models. Therefore, the advice and treatment the patients were given might have influenced the effect of fear of movement and distress on disability, potentially reducing the strength of the association between the candidate prognostic factors and disability outcomes [47].

## Conclusions

In patients with shoulder pain referred to secondary care we found that both higher fear of movement and emotional distress scores at baseline were associated with higher self-reported disability over the follow-up year, when adjusted for established prognostic factors. Our study indicates that fear of movement and emotional distress affect the prognosis in patients with shoulder pain and should be considered by clinicians and researchers working with patients with shoulder pain.

## Data Availability

The datasets used and/or analyzed during the current study are available from the corresponding author on reasonable request.

## Abbreviations

BMI: Body mass index
LMM: Linear mixed-effects models
NSD: Norwegian Social Science Data Service
QuickDASH: Quick Disability of the Arm and Shoulder
